# Polar extracts from (Tunisian) *Acacia salicina *Lindl. Study of the antimicrobial and antigenotoxic activities

**DOI:** 10.1186/1472-6882-12-37

**Published:** 2012-04-10

**Authors:** Jihed Boubaker, Hedi Ben Mansour, Kamel Ghedira, Leila Chekir Ghedira

**Affiliations:** 1 Laboratory of Cellular and Molecular Biology, Faculty of Dental Medicine, University of Monastir, Rue Avicenne, Monastir 5000, Tunisia; 2 Unity of Pharmacognosy/Molecular Biology, Faculty of Pharmacy, University of Monastir, Rue Avicenne, Monastir 5000, Tunisia; 3 Department of Cellular and Molecular Biology, Faculty of Dental Medicine, Rue Avicenne, 5000 Monastir, Tunisia

**Keywords:** *Acacia salicina*, Antigenotoxic activity, Antioxidant activities, Ames assay

## Abstract

**Background:**

Methanolic, aqueous and Total Oligomer Flavonoids (TOF)-enriched extracts obtained from the leaves of *Acacia salicina *'Lindl.' were investigated for antibacterial, antimutagenic and antioxidant activities.

**Methods:**

The antimicrobial activity was tested on the Gram positive and Gram negative reference bacterial strains. The Mutagenic and antimutagenic activities against direct acting mutagens, methylmethane sulfonate (MMS) and 4-nitro-o-phenylenediamine (NOPD), and indirect acting mutagens, 2-aminoanthracene (2-AA) and benzo[a]pyrene (B(a)P) were performed with *S. typhimurium *TA102 and TA98 assay systems. In addition, the enzymatic and nonenzymatic methods were employed to evaluate the anti-oxidative effects of the tested extracts.

**Results:**

A significant effect against the Gram positive and Gram negative reference bacterial strains was observed with all the extracts. The mutagenic and antimutagenic studies revealed that all the extracts decreased the mutagenicity induced by B(a)P (7.5 μg/plate), 2-AA (5 μg/plate), MMS (1.3 mg/plate) and NOPD (10 μg/plate). Likewise, all the extracts showed an important free radical scavenging activity towards the superoxide anion generated by the xanthine/xanthine oxidase assay system, as well as high Trolox Equivalent Antioxidant Capacity (TEAC), against the 2,2'-azino-bis(3-ethylbenzothiazoline-6-sulfonic acid) diammonium salt (ABTS)^+^• radical. TOF-enriched extract exhibited the highest protective effect against free radicals, direct acting-mutagen and metabolically activated S9-dependent mutagens.

**Conclusions:**

The present study indicates that the extracts from *A. salicina *leaves are a significant source of compounds with the antimutagenic and antioxidant activities, and this may be useful for developing potential chemopreventive substances.

## Background

Plants are rich source of natural products used for centuries to cure various diseases. The plant-derived medicines are based upon the premise that they contain natural substances that can promote health and alleviate illness. So, a retrospection of the healing power of plants and a return to natural substances are an absolute need of our time. The demonstration of the presence of natural products, such as polyphenols, alkaloids, flavonoids, coumarins and other secondary metabolites in medicinal plants will provide a scientific validation for the popular use of these plants [1].

*Acacia *(Fabaceae) is an evergreen tree that is native of Australia, but it is now widely distributed in the Mediterranean area. *Acacia *is a large genus comprising more than 700 species. The genus *Acacia *is frequently used for the treatment of various illnesses because of their reputed pharmacological effects; published informations indicate that *Acacia *has hypoglycemic [2], antibacterial [3], anti-inflammatory [4], cestocidal [5], spasmogenic and vasoconstrictor [6], antihypertensive and antispasmodic activities [7], anti-aggregation platelet effect [8], as well as an inhibitory effect against hepatitis C virus [9]. In Tunisian traditional medicine, the use of *Acacia *differs according to the species and according to the region. Based on informations gathered from traditional healers, herbalists, and inhabitants of rural south Tunisia, *Acacia salicina *has frequently been used as a the treatment of several diseases, such as the treatment of inflammatory diseases, as "febrifuge" to treat cancer, and as a fertility enhancer. In the south Tunisia, infusions prepared from fresh or dried leaves are taken orally, or alternatively, chopped fresh leaves are applied directly on inflamed sores. Traditional medical uses of *Acacia *in the north Tunisia are somewhat different [10].

Some *Acacia *species, and among them *Acacia salicina*, were described to be rich in tannins. Tannins obtained from *A. salicina *were reported to be responsible for the microbial activity [11].

Hence, in this paper we examined the antimicrobial, antimutagenic, and antioxidant activities of polar extracts obtained from *Acacia salicina *leaves. Our study revealed an interaction between the secondary metabolite composition of extracts, and each radical and/or bacterial strain used in the different assays.

## Results and discussion

### Antimicrobial activity

The antibacterial activity of the three tested *A. salicina *leaf extracts was evaluated on five pathogenic bacteria. Our results showed that these extracts exhibited various levels of antibacterial effect against all the tested bacterial strains. Minimum Inhibitory Concentration (MICs) values ranged from 0.0625 to over 10 mg/ml, and Minimum Bactericidal Concentration (MBCs) values ranged from 0.125 to more than 10 mg/ml. Generally, TOF extract displayed a strong activity against both Gram-negative and Gram-positive bacteria. The result of the antimicrobial activity is presented in Table 1.

**Table 1:** Antibacterial activity of *Acacia salicina *extracts, expressed as Minimum Inhibitory Concentration (MIC) and as Minimum Bactericidal Concentration (MBC)

	Gram positive organisms (mg/mL)				Gram negative organisms (mg/mL)					
	
	*S. aureus *ATCC25923		*E. faecalis *ATCC25922		*E. coli *ATCC 25922		*S. enteritidis* ATCC 13076		*S. typhimurium* NRRLB 4420	
	
**Extracts**^**a**^	MIC	MBC	MIC	MBC	MIC	MBC	MIC	MBC	MIC	MBC
Metanolic extract	1	2.5	2.5	5	> 10	> 10	2.5	5	2.5	7.5

Aqueous extract	1.25*	2.5	2.5	5	2.5	5	2.5	5	2.5	5

TOF extract	0.0625*	0.125*	0.25*	0.5*	> 10	> 10	0.25*	0.5*	0.125*	0.5*

Ampicilin b	0.0015	0.225	0.0025	0.125	0.006	0.275	0.0019	0.085	0.0039	0.26

* *P *< 0.05 compared to negative control without the tested extract by student test.

^a ^Values were expressed as means ± standard deviation of three experiments

^b^) Positive control

*Staphylococcus aureus *was the most susceptible bacterial species, followed by *Salmonella typhimurium*, then *Salmonella enteritidis *and *Enterococcus faecalis *and finally *Escherichia coli*, with MIC values of 0.062, 0.125, 0.250, 0.250 and > 10 mg/ml respectively. Compared to ampicillin, used as a positive control against *S. aureus *(0.225 mg/ml), the tested TOF extract was twice more active with MBC value of 0.125 mg/ml. *E. coli *was found to be the least sensitive strain to *A. salicina *extracts.

Compared to the other extracts, TOF extract was the most active one against all the tested bacterial strains. Its biological efficiency is probably related to the high amounts of flavonoids and polyphenolic compounds, in its chemical composition. We previously reported, that *A. salicina *extracts, particularly TOF extract, contains flavonoidic, polyphenolic and coumarinic compounds [12]. These families of compounds are reported to play a role in the prevention of colonisation by parasites, bacteria and fungi [13].

Our results indicate that Gram-positive bacteria are more sensitive to the antimicrobial effect of *A. salicina *extracts than Gram-negative ones. It is interesting to note that *A. salicina *extracts exhibited an antimicrobial activity, particularly towards organisms of interest to the medical field such as *Staphylococci*, *Enterococci *and *Salmonella*. In fact, *Salmonella *remains a primary cause of food poisoning worldwide, and massive outbreaks have been reported in recent years. The centre for disease control and prevention estimated that approximately 1.4 million cases of salmonellosis were annually reported in the United States [14]. The European Union reported more than 100.000 cases of salmonellosis [15]. In Tunisia, between 1978 and 1993, 1022 *Salmonella *strains were isolated: 578 in hospitals and 444 from the environment [16]. Some pathogenic *Salmonella *serotypes adapted to man, such as *S. typhimurium*, usually cause severe diseases such as enteric fever in humans. However, some pathogenic *Salmonella *serotypes, such as *S. enteritidis *or *S. typhimurium*, can infect a wide range of hosts and are termed ubiquitous. Likewise, foodborne illness resulting from the consumption of food contaminated with pathogenic bacteria, has been a vital concern to public health. *Salmonella *spp. and *E. coli *accounted for the largest number of outbreak cases and deaths.

### Antioxidant activities

#### Radical-Scavenging activity against ABTS^+^

The free radical scavenging capacity of *A. salicina *extracts was evaluated using the ABTS assay (Table 2). Decolorization of ABTS^+• ^reflects the capacity of antioxidant species to donate electrons or hydrogen atoms to inactivate this radical cation. A potential activity was noted at the different tested concentrations of all the studied extracts. The tested extracts seem to be more active than the positive control, trolox compound, as IC_50 _value obtained with trolox (0.76 mg/ml) was higher than IC_50 _value obtained with TOF, methanol and aqueous extracts (0.11, 0.39 and 0.24 mg/ml respectively). In fact, the tested extracts are complex mixtures of several compounds, in particular phenolic compounds with diverse chemical structures that determine various properties. The antioxidant effect of polyphenols against ABTS^+^. was reported earlier [17,18], similar to our observations in the current study. The reaction pattern consists of initial fast scavenging activity, where more active compounds react immediately with the radical. Products are formed, and together with the less reactive molecules, give a second slow reaction. The results obtained with our extracts corroborate this type of kinetic behaviour in all the samples and dilutions assayed. According to the result reported in the Table 2.

**Table 2:** Concentration-dependent ABTS free radical scavenging activity of *A. salicina *leaves extracts and standard antioxidant Trolox

**Extracts**^**b**^	Concentration (mg/ml)	**Inhibition (%)**^**a**^	TEAC (mM)	IC_50 _(mg/mL)
Aqueous extract	0.5	76 ± 1	2.19*	0.24*
			
	2.5	100 ± 2		
			
	4.5	100 ± 3		
			
	7.5	100 ± 5		
			
	9.5	100 ± 2		

TOF extract	0.5	100 ± 1	1.92*	0.11*
			
	2.5	100 ± 2		
			
	4.5	100 ± 2		
			
	7.5	100 ± 1		
			
	9.5	100 ± 4		

Methanolic extract	0.5	60.7 ± 4.3	1.65*	0.39*
			
	2.5	97.8 ± 3		
			
	4.5	99 ± 4		
			
	7.5	100 ± 2		
			
	9.5	100 ± 1		

Trolox^c^	0.5	22 ± 1	1	0.76
			
	0.625	32 ± 1		
			
	0.833	53.8 ± 2.5		
			
	1.25	65 ± 2		
			
	2.5	96.8 ± 2.5		

* *P *< 0.05 compared to negative control without the tested extract by student test.

^a ^Inhibition of absorbance at 734 nm relative to that of standart ABTS solution

^b ^Values were expressed as means ± standard deviation of three experiments

^c ^positive control

The TEAC values obtained with the different extracts reflect the relative ability of hydrogen or electron-donating antioxidants to scavenge the ABTS radical cation compared to that of Trolox (Table 2). When referring to TEAC values, TOF, aqueous and methanolic extracts revealed potent antioxidant capacities, with TEAC values of 1.92, 2.19 and 1.65 mM respectively. The values largely exceed the TEAC of the positive control Trolox (1 mM).

#### Effects on superoxide anion generating system

**Figure 1:**
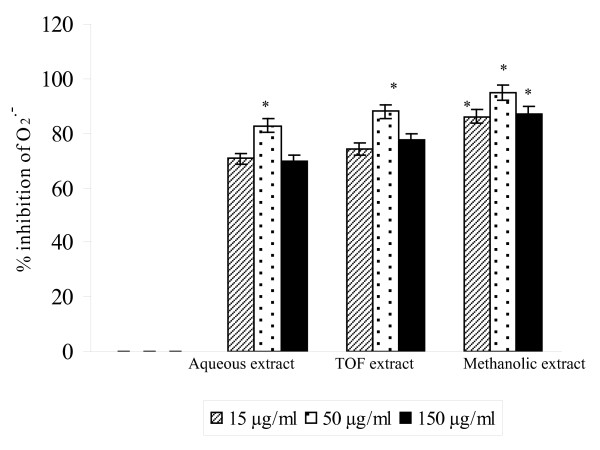
**Scavenging effects of extracts of *Acacia salicina *against X/XOD-generated superoxide free radicals (O_2_·^-^)**. *P<0.05.

The antioxidant activity of *A. salicina *leaf extracts was evaluated by the xanthine oxydase enzymatic system. The influence of *A. salicina *leaf extracts on XOD activity and/or the superoxide anions (O_2 _^•-^) enzymatically generated by this system, was evaluated *in vitro*. The results indicate that *A. salicina *extracts decreased significantly the XOD-generated superoxide radical with a maximum decrease at the concentration 50 μg/ml for each extract (Figure 1). In a previous work, these extracts were found to be able to decrease the uric acid produced by the xanthine/xanthine oxidase (X/XOD) reaction [12]. They were also, able to scavenge superoxide radical generated by the non-enzymatic system NBT/riboflavin. This clearly demonstrates that *A. salicina *extracts provoke both inhibition of XOD activity and scavenging of superoxide anion. These effects are probably mediated by active components in the extract.

Phenolic compounds present in TOF, methanolic and aqueous extracts [12] could probably contribute to the antioxidant potential of extracts, as described previously by Bouhlel et al [10].

### Mutagenic and antimutagenic activities

In experiments, prior to the mutagenicity study, it was ascertained that the different extracts added to the inductor bacteria do not influence their viability.

According to Ames et al. [19] and Marques et al. [20] a compound is classified as a mutagen if it is able to increase at least twice the number of revertants compared to spontaneous revertants. Based on this, most mutagenicity assays conducted with extracts were negative. None of the tested extracts produced a significant increase of his^+ ^revertant number of both *Salmonella typhimurium *TA 102 and TA 98 strains, in the absence of the S9 metabolizing system. In fact, mutation frequencies obtained with various concentrations of the tested samples do not change significantly when compared to spontaneous mutation frequencies. However, in the presence of S9, a mutagenic effect is observed with all the tested extracts, in the presence of *S. typhimurium *TA98 strain, at the two highest tested doses, and only with aqueous extract at the highest tested dose in the *S. typhimurium *TA 102 assay system. The result of antimutagenic activity in *S. typhimurium *TA98 assay system, is reported in Table 3.

**Table 3:** Effects of extracts from *A. salicina *on the mutagenicity induced by NOPD (10 μg/plate) and B(a)P (7.5 μg/plate) in *S. typhimurium *TA98 assay system respectively in the absence and in the presence of S9

		TA98			
		
		-S9		+S9	
**Extracts**	**Dose** **(μg/plate)**	**Nb** **Revertants/plate**	**% inhibition of mutagenesis**	**Nb Revertants/plate**	**% inhibition of mutagenesis**

Spontaneous	-	27 ± 6	-	36 ± 2	-

PC	-	723 ± 21	-	122 ± 11	-

Aqueous extract	50	235 ± 11	70.2*	66	65.1*
	
	250	127 ± 10	85.6*	49	84.8*
		
	500	87 ± 09	91.4*	50	83.7*

TOF extract	50	225 ± 08	71.6*	45	89.5*
	
	250	88 ± 06	91.2*	41	94.2*
	
	500	31 ± 03	99.4*	37	97.6*

Methanolic extract	50	207 ± 06	74.2*	65	66.3*
	
	250	122 ± 04	86.4*	61	71*
	
	500	67 ± 05	94.3*	41	95.2*

Positive control (PC): TA98/-S9, NOPD (10 μg/plate); TA98/+S9, B(a)P (7.5 μg/plate).

The result of mutagenic activity in *S. typhimurium *TA 98 and TA102 assays systems is reported in Table 4.

**Table 4:** Mutagenic activity of extracts from *A. salicina *by the *S. typhimurium *TA98 and TA102 assay systems in the presence and absence of the metabolic activation system (S9)

TA98				TA102	
**Extracts**	**Dose (μg/plate)**	**-S9**	**+S9**	**-S9**	**+S9**

Spontaneous	-	27 ± 6	36 ± 2	175 ± 10	235 ± 12

PC	-	723 ± 21	122 ± 11	1721 ± 24	652 ± 10

Aqueous extract	50	22 ± 2	42 ± 4	223 ± 11	387 ± 07
	
	250	24 ± 2	75 ± 5	234 ± 12	398 ± 12
	
	500	24 ± 3	85 ± 5	245 ± 09	477 ± 21

TOF extract	50	23 ± 3	54 ± 8	214 ± 08	345 ± 09
	
	250	25 ± 4	72 ± 10	221 ± 12	354 ± 05
	
	500	29 ± 3	102 ± 13	223 ± 11	377 ± 05

Methanolic extract	50	24 ± 4	44 ± 14	221 ± 10	276 ± 11
	
	250	31 ± 5	74 ± 13	242 ± 05	356 ± 12
	
	500	55 ± 7	95 ± 5	232 ± 08	455 ± 15

Positive control (PC): TA98/-S9, NOPD (10 μg/plate); TA102/-S9, MMS (10 μg/plate); TA98/+S9, B(a)P (7.5 μg/plate); TA102/+S9, 2-AA (5 μg/plate).

Besides, we envisaged the study of the antimutagenic activity of the same extracts toward four different mutagens, having diverse chemical structures and mode of actions, by using the Ames assay.

As shown in Table 3, all the extracts prepared from *A. salicina *were effective in reducing the mutagenicity induced by NOPD (10 μg/plate without S9) and MMS (2.5 mg/plate), a directacting mutagens, in respectively *S. typhimurium *TA 98 and TA 102 assay systems. All the tested extracts showed a clear dose-dependent response. Compared to methanolic and aqueous extracts, TOF extract was more effective against both NOPD and MMS direct mutagens. It reduces their mutagenicity by 99.4% and 93.5% respectively, at the highest tested dose (500 μg/plate). The possible mechanism of the potent protection of all the tested extracts against direct mutagens such as NOPD and MMS could be explained by the induction of the oxidative defence system and/or DNA repair enzymes, which are required to protect against oxidative-like mutations. Teel et al. [21] reported that the antimutagenic/anticarcinogenic activity of plants may be due to the interaction of the compounds with target DNA tissue, which, in turn, blocks the site(s) of DNA to electropholic attack by reactive mutagenic moieties. In our case, we speculate that the protective effect of the extracts against the tested mutagens is probably exerted by three different ways; firstly, the plant extracts may adsorb the mutagen in a way similar to the carcinogen adsorption which has been associated with chemical component; secondly, extracts could induce

DNA glicosylase enzymes which are capable of repairing alkylating DNA bases, and finally the reductive ability of the samples assessed in this study suggests that extracts were able to donate electrons to free radicals, making the radicals stable and unreactive [22].

Methanolic, aqueous and TOF extracts reduced the mutagenicity caused by the indirect mutagen B(a)P (7.5 μg/plate), a metabolically activated genotoxin, using the *S. typhimurium *TA 98 strain, in a dose dependent manner (Table 3). Our results, revealed that TOF extract was the more potent inhibitor of frame-shift mutations, due to the lack of a base pair in the GC-pair regions of gene D, induced by B(a)P in the *S. typhimurium *TA 98 assay system. When we tested at doses of 50, 250 and 500 μg/plate, all extracts, mixed with 2-AA, showed a toxic effect on *S. typhimurium *TA102 cell viability. This effect could be due to the formation of a complex between the mutagenic agent (2-AA) and extract components, inducing cell death at high concentration [22]. Therefore, decreased extract concentrations to 5, 10 and 25 μg/plate, did not influence bacterial viability. The tested extracts revealed an inhibitory effect against the mutagenicity induced by the 2-AA by, respectively, 32.6%, 44.6% and 32.6% at the lowest tested dose (5 μg/plate). The result of antimutagenic activity in *S. typhimurium *TA102 assay system, is reported in Table 5.

**Table 5:** Effects of extracts from *A. salicina *on the mutagenicity induced by MMS and 2-AA in *S. typhimurium *TA102 assay system respectively in the absence and in the presence of S9

TA102					
**Extracts**	**Dose (μg/plate)**	**-S9**	**+S9**		

		**Nb Revertants/plate**	**% inhibition of mutagenesis**	**Nb Revertants/plate**	**% inhibition of mutagenesis**

Spontaneous	-	175 ± 10	-	235 ± 12	-

PC	-	1721 ± 24	-	652 ± 10	-

Aqueous extract	5	NT	NT	600 ± 3	44.6*
	
	10	NT	NT	580 ± 8	17
	
	25	NT	NT	466 ± 5	12.4
	
	50	477	80.5*	T	-
	
	250	398	85.6*	T	-
	
	500	387	86.3*	T	-

TOF extract	5	NT	NT	516 ± 15	32.6*
	
	10	NT	NT	520 ± 17	31.6*
	
	25	NT	NT	619 ± 14	8
	
	50	377	86.9*	T	-
	
	250	354	88.4*	T	-
	
	500	276	93.5*	T	-

Methanolic extract	5	NT	NT	516 ± 9	32.6*
	
	10	NT	NT	520 ± 3	31*
	
	25	NT	NT	572 ± 9	20
	
	50	455	81.8*	T	-
	
	250	356	88.3*	T	-
	
	500	345	89*	T	-

*Nb *= number of revertant/plate ± SD; *PC *= Positive control TA102/-S9, MMS (10 μg/plate); TA102/+S9, 2-AA (5 μg/plate); *NT*: not tested, *T*: toxic

The inhibitory effects of *A. salicina *leaf extracts on the mutagenicity of both direct mutagens (NOPD, MMS) and metabolically activated mutagens (B(a)P, 2-AA), may be ascribed to flavonoid and tannin contents of TOF, methanolic and aqueous extracts [12]. We cannot, however, exclude the possibility of other compounds, with antimutagenic properties, participating to the antimutagenic effect of *A. salicina *extracts. The results of our experiments are consistent with the known antioxidant activities of flavonoids [23] and tannins [24]. Flavonoids are the most likely candidates, among the known compounds, to be present in the TOF-enriched, methanolic and aqueous extracts, involved in the antimutagenic activity of *A. salicina *extracts and in preventing oxidative lesions [25]. In this study, we used *S. typhimurium *TA102 strain, which is generally selected for specific detection of the oxidative damages [26]. Thus we suppose that the constituents *A. salicina *extracts should inhibit free radicals and ROS, produced by oxidation and redox-cycling of both B(a)P and 2- AA, and through reducing the activity of enzymes involved in B(a)P and 2-AA metabolisation. The extracts may both inhibit microsomal activation and directly protect DNA from the electrophilic B(a)P epoxide; 7,8-dihydroxy, 9,10-epoxy-7,8,9,10- tetrahydrobenzo[*a*]pyrene, a putative ultimate carcinogenic metabolite [27]. They may also protect DNA from the electrophilic *N*-hydroxy-2-aminoanthracene, a metabolite of 2-AA that interacts with DNA [28] as well as from other intermediates of the two aforementioned mutagens. In fact, several metabolic intermediates and ROS formed during microsomal enzyme activation are also capable of breaking DNA strands. The antioxidant activity expressed by *A. salicina *extracts may provide a common mechanism for inhibiting the mutagenicity of both B(a)P and 2-AA. However, the toxic effects obtained with the highest doses (500, 250 and 50 μg/plate) against *S. typhimurium *TA102 strain, when extracts were combined with mutagens, can be explained by the presence of molecules in these extracts which form, with the control mutagen, complexes with high bactericidal effect. In the presence of lower doses, probably, a weak number of molecules should react with 2-AA metabolites giving minor bactericidal complexes. No lethal effect, against *S. typhimurium *TA 102 strain is then detected.

We also noticed that methanolic, aqueous and TOF-enriched extracts were more efficient in reducing B(a)P mutagenicity than 2-AA mutagenicity. Curiously, these extracts exhibited both mutagenic and antimutagenic activities. We hypothesize that the presence of reactive intermediates resulting from both B(a)P and the tested extracts could result in their mutual neutralization (antagonist effects). These intermediates may form complexes preventing their penetration through the bacterial cell wall and thus inhibiting their mutagenic effects [29].

The chemical analysis of leaf extracts from *A. salicina*, harvested from the south east of Tunisia [12] revealed important differences from those obtained from *A. salicina *collected from the centre of Tunisia [10,29]. These differences may explain the different biological activities revealed by the two *A. salicina *ecotypifies

On the other hand, our study is in accordance with results reported by Mansour et al. [12] as far as we confirmed the antigenotoxic effect of *A. salicina *extracts described by these authors, who used a different prokaryotic assay i.e. the SOS chromotest in the presence of *E. Coli *PQ37, described by Quillardet and Hofnung [30]. However, some differences arised from evaluating O_2 _.^- ^scavenging capacity when comparing the antioxydant results of the present study and those reported by Ben Mansour *et al. *[12]. They revealed no scavenging effects against superoxyde anion. This could be explained by the different antioxydant assays used in each study. In fact, Ben Mansour *et al. *carried out a nonenzymatic O_2 _^. ^generating system to evaluate scavenging effects of *A. salicina *leaf extracts. Yet, we used in the present study the enzymatic X/XOD superoxide generating assay system to evaluate O_2 _^. ^scavenging activity of *A. salicina *extracts.

## Conclusion

The results we obtained demonstrate a possible interaction between secondary metabolites from each extract with the tested radicals, as well as strains used for the and with the mutagenicity assay. Polar extracts from (Tunisian) *A. salicina *leaves exhibited significant radical scavenging potential as well as antimicrobial and antimutagenic properties. This work paves the way for studying the effect of components from this medicinal plant, in the treatment of cancer cells by eventually inducing apoptotic death.

## Methods

### Chemicals

Methylmethane sulfonate (MMS), 2-aminoanthracene (2-AA), Benzo[a]pyrene (B(a)P), 4-nitro-o-phenylenediamine (NOPD), xanthine (X), xanthine oxidase (XOD), 6-hydroxy-2,5,7,8-tetramethylchroman-2-carboxylic acid (Trolox), and 2,2'-azino-bis(3-ethylbenzothiazoline-6-sulfonic acid) diammonium salt (ABTS) were obtained from Sigma Co (St. Louis, USA). Oxoid nutrient broth N°2, Agar-Agar, yeast extract, bactotryptone were purchased from Fluka (Buchs, Suisse), histidine and biotine from Difco (Bordeaux, France). Aroclor 1254 was purchased from Supelco (Isle d'Abeau Chesnes, France).

### Plant materials

*A. salicina *was collected from the Arid Region Institute (IRA) situated in the south east of Tunisia, in October 2003. Botanical identification was carried out by Pr. M. Chaib [31] (Department of Botany, Faculty of Sciences University of Sfax, Tunisia). A voucher specimen (AS-10.03) has been kept in the laboratory of Pharmacognosy, Faculty of Pharmacy of Monastir for future reference. The leaves were shade-dried, powdered, and stored in a tightly closed container.

### Extraction procedure

The aqueous extract was prepared from 100 g of powdered leaves by boiling in 200 ml water for 15 to 20 min. The extract was filtered, lyophilised, and the residue was dissolved in water.

In order to obtain an extract enriched in Total Oligomer Flavonoids (TOF), we macerated the powdered leaves in 1:2 (v:v) water/acetone for 24 hours with continuous stirring. The extract was filtered and the acetone was evaporated under low pressure in order to obtain an aqueous solution. The tannins were partially removed by precipitation, with an excess of NaCl, for 24 hours at 5°C, and the supernatant was recovered. The latter fraction was extracted with ethyl acetate, followed by concentration and precipitation with an excess of chloroform. The precipitate was separated and yielded the TOF-enriched extract.

Methanol extract was obtained from the powdered leaves with a soxhlet apparatus (4-hours extraction). This extract was concentrated to dryness and kept at 4°C.

### Bacterial strains

*Salmonella typhimurium *strains TA102 and TA98, a histidine-requiring mutants, were kindly provided by Pr. I. Felzenswab, (State University of Rio de Janeiro, Brazil), and maintained as described by Maron and Ames [32]. The genotypes of the test strains were checked routinely for their histidine requirement, deep rough (*rfa*) character and the presence of the R factor. They were stored at -80°C. *S. typhimurium *TA98 is a frame-shift sensitive strain which contains the *hisD3052 *mutation. It also contains the plasmid pKM 101, and the products of the *mucAB *genes on this plasmid enhance SOS mutagenesis [26]. *S. typhimurium *TA102 strain carries the nonsense mutation *hisG428 *on the multicopy (30 copies) plasmid pAQl, over a chromosomal *his *deletion (TA102). *HisG428 *gene contains an A:T base pair at the mutant site. Strains carrying this mutation can be reverted by damage at A:T base pairs and by damage at G:C base pairs as well as by 3, 6 or 9 bp deletions at the mutant site [26,33,34]. The majority of intragenic base substitutions were A:T- > T:A transversions. Other transitions and transversions were seen at lower frequencies [26]. Thus the *hisG428 *mutation can be reverted by all possible base pair changes as well as by deletions. *S. typhimurium *TA102 strain differs from TA98 strain and the other standard tester strains, by its intact excision repair (*uvrB+*) capacity, which facilitates detection of cross-linking agents [26].

### *In vitro *antimicrobial activity

The antimicrobial activity of *A. salicina *extracts was tested on the Gram-positive bacteria *Staphylococcus aureus *ATCC 25923 and *Enterococcus faecalis *ATCC 29212 as well as the Gram negative bacteria *Escherichia coli *ATCC 25922, *Salmonella enteretidis *ATCC 13076 and *Salmonella typhimurium *NRRLB 4420, using the microdilution method [35]. Overnight grown microbial suspensions were standardized to approximately 10^5 ^cells/mL [36]. The microdilution method was used to determine the Minimum Inhibitory Concentrations (MICs) of *A. salicina *extracts; 100 μL of microbial suspension containing, approximately 105 cells/mL, was added to 100 μl of the extract dilution (concentrations ranging from 62.5 μg/mL to 10 mg/ml in water). A set of tubes containing only microbial suspension served as the negative control. These serially diluted cultures were then incubated at 37°C for 24 hours. Subsequently, 10 μL of each culture was plated on substance-free Muller-Hinton agar plates and further incubated at 37°C for 24 hours. MIC was defined as the lowest concentration of plant extract that completely suppresses cell growth. Minimal Bactericidal Concentration (MBC) was defined as the lowest concentration of extract that kills 99.99% of the tested bacteria [37].

### Radical-scavenging activity on ABTS^+•^

An improved ABTS radical cation decolorization assay was used. It involves the direct production of the blue/green ABTS^+• ^chromophore through the reaction between ABTS and potassium persulfate. Addition of antioxidants to the preformed radical cation reduces it to ABTS, to an extent and on a timescale depending on the antioxidant activity, the concentration of the antioxidant and the duration of the reaction [38]. ABTS was dissolved in water to a 7 mM concentration. ABTS^+• ^was produced by reacting ABTS stock solution with 2.45 mM potassium persulfate (final concentration) and allowing the mixture to stay in the dark at room temperature for 12 to 16 hours before use. The ABTS solution was diluted with ethanol to an absorbance of 0.7 (± 0.02) at 734 nm. In order to measure the antioxidant activity of extracts, 10 μl of each sample at various concentrations (0.5, 2.5, 4.5, 7.5 and 9.5 mg/mL), were added to 990 μl of diluted ABTS solution and the absorbance was recorded every 1 min. We stopped the kinetic reaction after 30 min. Each concentration was analysed in triplicate. The percentage decrease of absorbance at 734 nm was calculated for each point and the antioxidant capacity of the test compounds was expressed as inhibition percent. IC50 value (concentration required to reduce ABTS^+• ^by 50%) was calculated from a regression analysis. Trolox is used as a comparison standard for the determination of the antioxidant activity of a compound. The results are also reported as Trolox equivalent antioxidant capacity (TEAC), which is the molar concentration of Trolox giving the same percentage decrease of ABTS absorbance, as 1 mg/ml of the antioxidant testing extract, at a specific time point [38].

### Superoxide anion Scavenging Activity

The superoxide radical (O_2_^•^) is a highly toxic species that is generated by numerous biological and photochemical reactions via the Haber-Weiss reaction. It can generate the hydroxyl radical, which reacts with DNA bases, amino acids, proteins, and polyunsaturated fatty acids, and produces toxic effects. The toxicity of the superoxide radical could also be due to the perhydroxyl intermediates (HO_2_^•^) that react with polyunsaturated fatty acids. Finally, superoxide may react with NO to generate peroxynitrite, which is known to be toxic towards DNA, lipids and proteins.

### Xanthine oxidase

In our study, superoxide anion was generated by an enzymatic X/XOD assay system. The enzyme xanthine oxidase catalyses the oxidation of xanthine to uric acid. During this reaction, molecular oxygen acts as an electron acceptor, producing superoxide radicals according to the following equation:

$$\text{Xanthine}+{\text{O}}_{\text{2}}\overset{\text{Xanthine}\;\text{oxidase}}{\underset{}{\to }}\text{Uric}\;\text{acid}+{\text{O}}_{\text{2}}+{\text{H}}_{2}+{\text{H}}_{2}{\text{O}}_{2}$$

The superoxide anion scavenging activity was detected spectrophotometrically with the nitrite method described by Oyangagui [39] with some modifications introduced by Hu et al. [40] and Russo et al. [41]. Briefly, the assay mixture consisted of 100 μL of the tested compound solution, 200 μL of xanthine (final concentration 50 μM) as the substrate, hydroxylamine (final concentration 0.2 mM), 200 μL of Ethylenediaminetetraacetic acid (EDTA) (0.1 mM) and 300 μL of distilled water. The reaction was initiated by adding 200 μl of XOD (11 mU) dissolved in phosphate buffer (KH_2_PO_4 _20.8 mM, pH 7.5). The assay mixture was incubated at 37°C for 30 min. The reaction was stopped by adding 0.1 mL of HCl 0.5 M. Another control solution without the tested compound was prepared in the same manner as the assay mixture, to measure the total uric acid production (100%). To detect the superoxide scavenging activity, 2 mL of the colouring reagent consisting of sulphanilic acid solution (final concentration 300 μg/mL), N-(1-naphtyl) ethylenediamine dihydrochloride (final concentration 5 μg/mL) and acetic acid (16.7%, v/v) were added. This mixture was allowed to rest for 30 min at room temperature and the absorbance was measured spectrophotometrically at 550 nm. The absorbance was measured against a blank solution prepared as described above, but replacing XOD with buffer solution. The dose-effect curve for each test compound was linearized by a regression analysis and used to derive the IC50 values.

### Activation mixture

The S9 microsome fraction was prepared from rats treated with Aroclor 1254 [32]. The components of S9 mix were 1 mL of salt solution; 0.25 ml of 1 M glucose 6 phosphate; 2 ml of 0.1 M NADP; 25 ml of 0.2 M sodium phosphate buffer, pH 7.4; 7 mL of S9 microsome fraction and 14.75 mL H_2_O. The S9 mix was prepared freshly for each assay [32]. Protein concentration of S9 was determined using protein BioRad assay [42], it was found to be 12.3 mg/mL.

### *Salmonella-*microsome assay

The mutagenicity assay with *Salmonella typhimurium *was performed as described by Maron and Ames [32]. The experiments were performed with and without an exogenous metabolic system, the S9 fraction in S9 mix. We added 100 μL of bacterial exponential-phase culture and 500 μL of S9 mix for assay with S9, or 500 μL of sodium phosphate buffer (0.2 M, pH 7.4 for assay without S9) to 2 ml aliquots of top agar (supplemented with 0.5 mM L-histidine and 0.5 mM d-biotin), containing 100 μL of different concentrations of each tested extract. The resulting complete mixture was poured on minimal agar plates prepared as described by Maron and Ames [32]. The plates were incubated at 37°C for 48 hours and the revertant bacterial colonies of each plate were counted. The negative and positive control cultures gave numbers of revertants per plate that were within the normal limits found in the laboratory. An extract was considered mutagenic if the number of revertants per plate was at least doubling in *S. typhimurium *TA102 and TA98 strains over the spontaneous revertant frequencies [19,20]. The appropriate positive controls accepted for the Ames test were applied; these controls were selected according to the type of strain used, and the presence or absence of the S9 mix.

Data were collected with a mean ± standard deviation of three plates (*n *= 3).

### Antimutagenicity testing

The test was performed using the preincubation method to 0.5 ml of S9 mixture when using the indirect mutagens 2-AA (5 μg/plate) and B(a)P (7.5 μg/plate), or 0.5 mL of phosphate buffer (when using the direct mutagens MMS (10 μg/plate) and NOPD (10 μg/plate)). We added 0.1 mL of the test compounds (50 μL of mutagen and/or 50 μL of test compound) and 0.1 mL of bacterial culture (prepared as described in mutagenicity test). After vortexing gently and preincubating at 45°C for 30 min, 2 ml of top agar supplemented with 0.05 M Lhistidine and D-biotine were added to the mixture and vortexed for 3 s. The resulting entire was overlaid on a minimal agar plate. The plates were incubated at 37°C for 48 h and the revertant bacterial colonies on each plate were counted. The inhibition rate of mutagenicity (%) was calculated relative to those in the control group with the mutagen by the following formula: percent inhibition (%) = [1 - ((number of revertants on test plates - number of spontaneous revertants)/(number of revertants on positive control plates - number of spontaneous revertants))] × 100. Each dose was tested in triplicate.

### Statistical analyses

Data were collected and expressed as the mean ± standard deviation of three independent experiments and analyzed for statistical significance from control. The data were tested for statistical differences by student test. The criterion for significance was set at p < 0.05.

## Abbreviations

A. salicina: Acacia salicina; B(a)P: Benzo[a]pyrene; rfa: Deep rough; E. faecalis: Enterococcus faecalis; E. coli: Escherichia coli; MMS: Methylmethane sulfonate; MIC: Minimum Inhibitory Concentrations; MBC: Minimal bactericidal concentration; TOF: Total Oligomer Flavonoids; TEAC: Trolox Equivalent Antioxidant Capacity; S. typhimurium: Salmonella typhimurium; S. aureus: Staphylococcus aureus; S. entretidis: Salmonella entretidis; S. typhimurium: Salmonella typhimurium; X/XOD: Xanthine/xanthine oxidase; X: Xanthine; XOD: xanthine oxidase; 2-AA: 2-aminoanthracene; NOPD: 4-nitro-o-phenylenediamine; ABTS: 2,2'-azino-bis(3-ethylbenzothiazoline-6-sulfonic acid) diammonium salt; Trolox: 6-hydroxy-2,5,7,8-tetramethylchroman-2-carboxylic acid.

## Competing interests

The authors declare that they have no competing interests.

## Authors' contributions

**JB**: Was responsible for the conception and design, testing and data acquisition, analysis and data interpretation and drafted the manuscript. **HBM: **Was responsible for the conception and design, testing and data acquisition, analysis and data interpretation and drafted the manuscript. **The two authors have equally contributed to this work. KG: **made substantial contribution to conception and revised it critically for important intellectual content. **LCG: **made substantial contribution to conception and revised it critically for important intellectual content. All authors read and approved the final manuscript.

## Pre-publication history

The pre-publication history for this paper can be accessed here:

http://www.biomedcentral.com/1472-6882/12/37/prepub

## References

[B1] Swayamjot K Husheem M Saroj A Pirkko LH Subodh-Kumar K The in vitro cytotoxic and apoptotic activity of Triphala-an Indian herbal drug J Ethnopharmacol 2005 97 15 20 10.1016/j.jep.2004.09.050 15652269

[B2] Wadood A Wadood N Wahid-Shah SA Effects of *Acacia arabica *and *Caralluma edulis *on blood glucose levels of normal and alloxan diabetic rabbits J Pak Med Assoc 1989 39 208 212 2509753

[B3] Sotohy SA Sayed AN Ahmed MM Effect of tannin-rich plant (*Accacia nilotica*) on some nutritional and bacteriological parameters in goats Deutsche Tierarztliche Wochenschrift 1997 104 432 435 9394540

[B4] Dafallah AA Al-Mustapha Z Investigation of the anti-inflammatory activity of *Acacia nilotica *and *Hibiscus sabdariffa* Am J Chinese Med 1996 24 263 269 10.1142/S0192415X96000323 8982438

[B5] Ghosh NK Babu SP Sukul NC Ito A Cestocidal activity of *Acacia auriculiformis* J of Helmintol 1996 70 171 172 10.1017/S0022149X00015340 8960214

[B6] Amos S Akah PA Odukwe CJ Gamaniel KS Wambede C The pharmacological effects of an aqueous extract from *Acacia nilotica *seeds Phytother Res 1999 13 683 685 10.1002/(SICI)1099-1573(199912)13:8<683::AID-PTR534>3.0.CO;2-X 10594939

[B7] Gilani AH Shaheen F Zaman M Janbaz KH Shah BH Akhtar MS Studies on hypertensive and antispasmodic activities of methanol extract of *Acacia nilotica *pods Phytother Res 1999 14 510 516 10.1002/(sici)1099-1573(199912)13:8<665::aid-ptr563>3.0.co;2-t 10594935

[B8] Shah BH Safdar B Virani SS Nawaz Z Saeed SA Gilani AH The antiplatelet aggregatory activityof *Accacia nilotica *is due to blockage of calcium influx through membrane calcium channels General Pharmacol 1997 29 251 255 10.1016/S0306-3623(96)00413-2 9251908

[B9] Hussein G Miyashiro H Nakamura N Hattori M Kakiuchi N Shimotohno K Inhibitory effects of Sudanese medicinale plant eextracts on hepatitis C virus (HCV) Phytother Res 2000 14 510 516 10.1002/1099-1573(200011)14:7<510::AID-PTR646>3.0.CO;2-B 11054840

[B10] Bouhlel I Valenti K Kilani S Skandrani I Sghaier MB Antimutagenic, antigenotoxic and antioxidant activities of *Acacia salicina *extracts (ASE) and modulation of cell gene expression by H_2_O_2 _and ASE treatment TIV 2008 2008 22 1264 1272 10.1016/j.tiv.2008.04.008 18515041

[B11] Getachew G Makkar HP Becker Tannins in tropical browses: effects on in vitro microbial fermentation and microbial protein synthesis in media containing different amounts of nitrogen J Agric Food Chem 2000 48 3581 3588 10.1021/jf990740v 10956154

[B12] Ben-Mansour H Boubaker J Bouhlel I Mahmoud A Bernillon S Ben-Chibani J Ghedira K Chekir-Ghedira L Antigenotoxic activities of crude extracts from *Acacia salicina *leaves Environ Mol Mutagen 2007 48 58 66 10.1002/em.20265 17177209

[B13] Chiang LC Chiang W Liu MC Lin CC In vitro antiviral activities of *Caesalpinia pulcherrima *and its related flavonoids J Antimicrob Chemother 2003 52 194 198 10.1093/jac/dkg291 12837746

[B14] Mead PS Slutsker L Dietz V McCaig LF Bresee JS Shapiro C Griffin PM Tauxe RV Food-related illness and death in the United States Emerg Infect Dis 1999 5 607 625 10.3201/eid0505.990502 10511517 PMC2627714

[B15] O'Brien SJ de Valk H *Salmonella*-"old" organism, continued challenges! Eurosurveillance 2003 8 29 31 12631971

[B16] Dhidah M Trabelsi A Mazoughi R Dhidah L Boujaafar N Jeddi M *Salmonella *and salmonelloses in the region of Sousse (Tunisia) Microbiol Hyg Alim 1995 19 3 7

[B17] Van der Berg R Haenen GRMM Van der Berg H Van den Vijgh W Bast A The predictive value of the antioxidant capacity of structurally related flavonoids using the Trolox equivalent antioxidant capacity (TEAC) assay Food Chem 2000 70 391 395 10.1016/S0308-8146(00)00092-3

[B18] Villaño D Fernãndez-Pachõn MS Troncoso AM Garciã-Parrilla MC The antioxidant activity of wines determined by the ABTS+• method: influence of sample dilution and time Talanta 2004 64 501 509 10.1016/j.talanta.2004.03.021 18969632

[B19] Ames BN McCann J Yamasaki E Methods for detecting carcinogens and mutagens with the *Salmonella*/mammalian microsome mutagenicity test Mut Res 1975 31 347 364 768755 10.1016/0165-1161(75)90046-1

[B20] Marques RCP de Medeiros SRB da Silva Dias C Barbosa-Filho JM Agnez-Lima LF Evaluation of the mutagenic potential of yangambin and of the hydroalcoholic extract of *Ocotea duckei *by the Ames test Mutat Res 2003 536 117 120 12694751 10.1016/s1383-5718(03)00040-8

[B21] Teel RW Ellargic acid binding to DNA as a possible mechanism for its antimutagenic and anticarcinogenic action Cancer Letter 1986 30 329 336 10.1016/0304-3835(86)90058-3 3697951

[B22] Boubaker J Skandrani I Bouhlel I Ben Sghaier M Neffati A Ghedira K Chekir-Ghedira L Mutagenic, antimutagenic and antioxidant potency of leaf extracts from *Nitraria retusa* Food and Chem Toxicol 2010 48 2283 2290 10.1016/j.fct.2010.05.061 20510330

[B23] Vaya J Mohmod S Goldblum A Aviram M Volkavor N Shaalam A Musa R Tamir S Inhibition of LDL oxidation by flavonoïds in relation to their structure and calculated enthalpy Phytochem 2003 62 89 99 10.1016/S0031-9422(02)00445-4 12475624

[B24] Zoran M Dorde M Nada K Polyphenol contents and antioxidant activity of *Maydis stigma *extracts Biores. Technol 2005 96 873 877 10.1016/j.biortech.2004.09.006 15627557

[B25] Edenharder R Grunhage D Free radical scavenging abilities of flavonoids as mechanism of protection against mutagenicity induced by *test*-butyl hydroperoxide or cumene hydroperoxide in *Salmonella typhimurium *TA 102 Mutat Res 2003 540 1 18 12972054 10.1016/s1383-5718(03)00114-1

[B26] Levin DE Hollstein M Christman MF Schwiers EA Ames BN A new *Salmonella *tester strain (TA102) with A.T base pairs at the site of mutation detects oxidative mutagens Proc Natl Acad Sci USA 1982 79 7445 7449 10.1073/pnas.79.23.7445 6760198 PMC347356

[B27] Harris CC Vähäkangas K Newman MJ Trivers GE Shamsuddin A Sinopoli N Mann DL Wright WE Detection of benzo[*a*]pyrene diol epoxide-DNA adducts in peripheral blood lymphocytes and antibodies to the adducts in serum from coke oven workers Proc Natl Acad Sci USA 1985 82 6672 6676 10.1073/pnas.82.19.6672 2413443 PMC391272

[B28] Horn RC Ferrao VVM Antimutagenic activity of extracts of natural substances in the *Salmonella *microsome assay Mutag 2003 18 113 118 10.1093/mutage/18.2.113 12621065

[B29] Bouhlel I Ben Mansour H Limem I Ben Sghaier M Mahmoud A Ben Chibani J Ghedira K Chekir-Ghedira L Screening of antimutagenicity via antioxidant activity in different extracts from the leaves of *Acacia salicina *from the center of Tunisia Environm Toxicol Pharmacol 2007 23 56 63 10.1016/j.etap.2006.07.001 21783737

[B30] Quillardet PQ Hofnung M The SOS Chromotest colorimetric bacterial assay for genotoxins: Procedures Mutat Res 1985 147 65 78 3923333 10.1016/0165-1161(85)90020-2

[B31] Chaib M Boukhris M Flore succinct et illustré des zones arides et sahariennes de Tunisie 1998 43 44

[B32] Maron DM Ames BN Revised methods for the *Salmonella *mutagenicity test Mutat Res 1983 113 173 215 6341825 10.1016/0165-1161(83)90010-9

[B33] Hartman PE Ames BN Roth JR Branes WN Levin DE Target sequences for mutagenesis in *Salmonella *histidine-requiring mutants Environ Mol Mutagen 1986 8 631 641 10.1002/em.2860080414 3525139

[B34] Koch WH Henrikson EN Cebula TA Molecular analyses of *Salmonella *hisG428 ochre revertants for rapid characterization of mutational specificity Mutagenesis 1996 11 341 348 10.1093/mutage/11.4.341 8671758

[B35] Hayder N Abdelwahed A Kilani S Ammar RB Mahmoud A Ghedira K ChekirGhedira L Anti-genotoxic and free-radical scavenging activities of extracts from (Tunisian) *Myrtus communis* Mut Res 2004 564 89 95 15474415 10.1016/j.mrgentox.2004.08.001

[B36] Cremieux A Fleurette J Methods of testing disinfectants. In disinfection, sterilization and preservation 1991 4 Lea and Febiger, Philadelphia and London 1009 1027

[B37] Ben Ammar R Kilani S Bouhlel I Skandrani I Naffeti A Boubaker J Ben Sghaier M Bhouri W Mahmoud A Chekir L Ghedira K Antibacterial and cytotoxic activities of extracts from (Tunisian) *Rhamnus alaternus *(Rhamnaceae) Ann Microbio 2007 57 453 460 10.1007/BF03175089

[B38] Re P Proteggente R Pannala N Yang A Rice-Evans CMA Antioxidant activity applying an improved ABTS radical cation decolorization assay Free Radic Biol Med 1999 26 1231 1237 10.1016/S0891-5849(98)00315-3 10381194

[B39] Oyangagui Y Reevaluation of assay methods and establishment of kit for superoxide dimutase activity Analy Biochem 1984 142 290 296 10.1016/0003-2697(84)90467-6 6099057

[B40] Hu JP Calomme M Lasure A De Bruyne T Pieters L Vlietinck A Vanden Berghe DA Structure activity relationship of flavonoids with superoxide scavenging activity. *Biol* Trace Elem Res 1995 47 327 331 10.1007/BF02790134 7779566

[B41] Russo A Cardile V Lombardo L Vanella L Vanella A Garbarino JA Antioxidant and antiproliferative action of methanolic extract of Geum Quellyon sweet roots in human tumor cell lines Journal of Ethnopharma 2005 100 323 332 10.1016/j.jep.2005.03.032 15941635

[B42] Bradford MM A Rapid and Sensitive Method for the Quantitation of Microgram Quantities of Protein Utilizing the Principle of Protein-Dye Binding Annals Biochemestry 1976 72 248 254 10.1016/0003-2697(76)90527-3 942051

